# Intimate partner violence, traumatic brain injury and long-term mental health outcomes in midlife: the Drake IPV study

**DOI:** 10.1136/bmjment-2024-301439

**Published:** 2025-06-09

**Authors:** Natalie D Jenkins, Craig W Ritchie, Karen Ritchie, Graciela Muniz Terrera, William Stewart, on behalf of the PREVENT Dementia Investigators

**Affiliations:** 1 University of Glasgow, Glasgow, UK; 2 Department of Medicine, University of St Andrews, St Andrews, UK; 3 INM Institut de Neurosciences de Montpellier, Montpellier, France; 4 Department of Social Medicine, Ohio University Heritage College of Osteopathic Medicine, Athens, Ohio, USA; 5 Department of Neuropathology, Queen Elizabeth University Hospital, Glasgow, UK; 6 University of St Andrews, St Andrews, UK

**Keywords:** depression, anxiety disorders, depression & mood disorders

## Abstract

**Background:**

Approximately 30% of women experience intimate partner violence (IPV) in their lifetime, often with traumatic brain injury (TBI) exposure. Nevertheless, there has been limited research exploring lifelong brain health outcomes following IPV with TBI. To address this, we investigated the relationship between IPV, TBI and midlife mental health outcomes within an observational cohort study.

**Methods:**

PREVENT Dementia is a cohort study with participants recruited aged 40–59 years for longitudinal measures of brain health. Participants reporting histories of IPV-related physical abuse (IPV-PA) at study recruitment were identified and compared with control participants with no IPV-PA exposure regarding histories of TBI and prevalence of lifetime and ongoing mental health outcomes using standardised assessments.

**Results:**

Among 632 participants, 90 (14%) reported IPV-PA history. Compared with unexposed participants, history of IPV-PA was associated with higher TBI exposure, together with higher lifetime and ongoing diagnoses of depression, anxiety and sleep disorders, and post-traumatic stress disorder (PTSD) symptomology. Notably, the risk of ongoing and concurrent midlife mental health disorders remained despite IPV-PA exposure having ceased on average 27 years before assessment. History of TBI in individuals with IPV was associated with increased risk of ongoing PTSD symptomology and concurrent mental health outcomes.

**Conclusions:**

Our data confirm high TBI exposure among individuals with a history of IPV-PA, while also demonstrating that this population shows higher rates of ongoing adverse mental health outcomes in midlife, often decades after abuse. This work underlines the prevalence of IPV-PA and the necessity to consider TBI exposure and long-term brain health outcomes among this population.

**WHAT IS ALREADY KNOWN ON THIS TOPIC**

- Traumatic brain injury (TBI) is associated with adverse brain health outcomes, including neurodegenerative disease and neuropsychiatric disorders.
- The global prevalence of intimate partner violence (IPV) is high (30% of women), with injuries most frequently reported to the head, face and neck, often multiple.
- There is limited research exploring long-term brain health outcomes following IPV-related TBI, particularly in midlife, where we now recognise depression as a midlife risk factor for dementia.

**WHAT THIS STUDY ADDS**

- This study examined 632 participants from a population-based, longitudinal cohort study, PREVENT Dementia; 90 (14%) of whom report a history of IPV-related physical abuse (IPV-PA).
- IPV-PA was associated with higher risk of ongoing clinical diagnoses of depression, anxiety and post-traumatic stress disorder symptomology, as well as concurrent mental health outcomes.
- Ongoing diagnoses remained higher in midlife in individuals reporting IPV-PA, despite abuse ending many years prior to assessment.

**HOW THIS STUDY MIGHT AFFECT RESEARCH, PRACTICE OR POLICY**

- The study underlines the necessity to consider long-term brain health outcomes among individuals with histories of exposure to intimate partner violence.

## Introduction

 There is a widespread recognition that a history of traumatic brain injury (TBI) is associated with increased risk of a range of adverse lifelong brain health outcomes, including dementia^1^ and neuropsychiatric disorders.^2^,^4^ Up to 30% of women will experience physical or sexual intimate partner violence (IPV) in their lifetime, making it the most common form of violence globally.^5^ A frequent component of IPV is blows to the face, head and neck, often multiple,^6 7^ leading to some estimates suggesting there are more women living with histories of IPV-related TBI than contact sports athletes with histories of repetitive head impact (RHI) or TBI exposure.^8^ Nevertheless, in contrast to our rapidly evolving understanding of lifelong brain health outcomes related to contact sports participation, comparatively little is known regarding brain health consequences associated with IPV-related TBI.

The WHO defines IPV as ‘*any behaviour within an intimate relationship that causes physical, psychological or sexual harm to those in the relationship*’.^9^ This can include acts of physical or sexual violence, emotional abuse and controlling behaviours. While both men and women can be subject to IPV, rates are considerably higher among women.^10^ Furthermore, women are typically exposed to more severe IPV-associated violence than men,^11^ with the overwhelming majority subject to head and facial injuries associated with high exposure to TBI.^6^ Indeed, where data are available, between 27% and 100% of women with a history of IPV report histories of TBI.^10^ Nevertheless, despite its remarkably high prevalence, little is known of the lifelong brain health consequences associated with IPV-related RHI/TBI exposure.

The relationship between TBI and risk of neurodegenerative disease (NDD) has long been considered,^1^ with recent reporting of the link between contact sport-related TBI/RHI and increased NDD risk drawing fresh attention to this association.^12^ In addition to NDD, however, a range of neuropsychiatric outcomes have been documented following TBI.^2^,^4^ Thus, beyond NDD, TBI is reported to be associated with increased risk of depression, anxiety, disinhibition and suicide.^3 4^ With respect to IPV-related TBI, individuals exposed to it report a higher risk of a range of conditions, including depression, anxiety and post-traumatic stress disorder (PTSD).^13^,^15^ However, these studies generally focus on relatively short-term outcomes, typically recruit participants through women’s shelters, and are often limited by a lack of data regarding IPV history.^13^,^15^ The specific relationship between IPV-related TBI and delayed neuropsychiatric outcomes in midlife in a general population setting has not been explored. As such, there is a need to consider the lifelong mental health consequences of IPV-associated physical abuse (PA) and TBI.

In this context, we hypothesise that in a midlife, general population cohort recruited for studies of wider brain health, a history of IPV-related PA (IPV-PA) will be associated with evidence of adverse mental health outcomes. Furthermore, adverse mental health outcomes will be exacerbated in those with IPV history and TBI. To address these hypotheses, we leveraged comprehensive data available within the established PREVENT Dementia programme^16^ to investigate the relationship between IPV, TBI and indices of brain health in midlife.

## Methods

### Study Design, Participants & Inclusion Criteria

The study sample was drawn from the prospective, population-based, observational cohort study PREVENT Dementia. The full protocol and participant baseline data are described in detail elsewhere.^16^ In brief, 700 participants aged between 40 and 59 years without a diagnosis of an NDD at baseline were recruited to the study between 2014 and 2020. Participants were recruited from five sites across the UK and Ireland (Cambridge, Dublin, Edinburgh, London and Oxford). The current study includes cross-sectional analyses of baseline data.

### Intimate partner violence

Lifetime history of PA was assessed using the Lifestressor Checklist Revised, a 30-item questionnaire that assesses traumatic or stressful life events.^17^ For each of the 30 events, participants are asked how old they were at onset and end of exposure, where applicable. Participants were categorised as having been exposed to IPV-PA if they responded ‘yes’ to the questions *“Before age 16, were you ever abused or physically attacked (not sexually) by someone you knew (for example, a parent, boyfriend, or husband, hit, slapped, choked, burned, or beat you up)?”* or *“After age 16, were you ever abused or physically attacked (not sexually) by someone you knew (for example, a parent, boyfriend, or husband hit, slapped, choked, burned, or beat you up)?”*. Duration of IPV-PA was calculated from age of onset to age at last exposure. Participants who reported PA solely before age 16 years (ie, childhood) were removed from analyses.

### Brain injury

Lifetime history of brain injury was assessed using the Brain Injury Screening Questionnaire (BISQ), a structured self-report questionnaire that provides contextual cues to aid recall of remote head injury.^18^ The BISQ has been extensively validated in numerous community-based settings and is among tools recommended by the National Institutes of Neurological Disorders and Stroke Common Data Elements initiative and Enhancing NeuroImaging Genetics through Meta-Analysis (ENIGMA) Consortium Intimate Partner Violence Working Group for assessing lifetime TBI exposure.^6 19^ For each situational cue endorsed, participants were asked to record the number of blows to the head and, if so, whether they experienced any episodes of loss of consciousness (LOC) or were dazed and confused (DAC) following a blow to the head, the number of times this happened and the duration of each episode.

### Mental health

Lifetime clinical diagnoses of depression, anxiety, sleep disorder, mood disorders and psychotic disorders were self-reported by clinical interview with a study physician. Participants were asked if they had ever been clinically diagnosed with these conditions in their lifetime, along with the date of onset of diagnosis and whether the disorder was currently active and ongoing. PTSD symptomology was recorded using the LifeStressor Checklist Revised detailed above, which records the perceived level of danger at the time of the event and subsequent impact on everyday life within the past 12 months for each of the 30 traumatic life events. This allows calculation of Diagnostic and Statistical Manual of Mental Disorders, Fourth Edition (DSM-IV) criterion A for PTSD symptomology for each endorsed event, distinguishing serious from minor stressors.^17^ Lifetime PTSD symptomology was binary coded based on the presence/absence of any life event meeting DSM IV criteria A for PTSD. Ongoing/Active PTSD symptomology was binary coded (present/absent), where PTSD symptomology was reported to have had ‘some’ to ‘extreme’ effect on everyday life within the past 12 months.

### Statistical analysis

Group differences in sociodemographic characteristics were analysed using χ^2^ test for categorical variables, and analysis of variance (ANOVA) or Mann-Whitney U test (where assumptions of ANOVA were violated) for continuous variables. Multivariable linear and binary logistic regression models were used to examine the association between exposure to IPV-PA (IPV-PA/no exposure) and mental health outcomes, while controlling for age, self-reported gender (all either male or female), years of education and TBI with LOC (yes/no). Models of depression, anxiety and sleep disorders were also controlled for PTSD symptomology (yes/no). Mood and psychotic disorders were not modelled individually owing to small numbers of participants endorsing these conditions (fewer than 10 participants per group). The number of mental health conditions (anxiety, depression, sleep disorders, mood disorders, psychotic disorders and PTSD symptomology) between groups was modelled using Poisson regression, while controlling for age, gender, years of education and TBI with LOC. Finally, in individuals with exposure to IPV-PA, multivariable linear and binary logistic regression models were used to examine the association between mental health outcomes and characteristics of abuse including duration of abuse in years (continuous), years since abuse ended (continuous), TBI with LOC and age. All analyses were conducted in R (V.4.0.3), with statistical significance set at two-sided p<0.05.

## Results

### Sociodemographic characteristics

After removal of 59 participants reporting PA solely before age 16 years and nine with incomplete exposure data, a total of 632 participants were available for analysis. Of these, 90 (14.24%) reported a history of IPV-PA, with an average age at onset of 20.70±11.80 (mean±SD) years and a duration of exposure of 6.57±7.83 years. At assessment, the median age of participants with IPV-PA history was 53 (range 40–59) years, with a reported 27 (0–41) years since IPV-PA exposure ceased. Compared with participants without a history of IPV-PA, those with IPV-PA exposure were more often female (p<0.001; χ^2^) and APOEε4 carriers (p=0.03) but were less likely to report a parental history of dementia (p=0.03). There were no differences between groups in age at assessment, years of education or alcohol use (table 1).

**Table 1:** Sociodemographic characteristics of participants

	No exposure	IPV-PA	P value
N (%)	542 (85.76)	90 (14.24)	–
Median age, years (IQR)	52.00 (8.00)	53.00 (9.00)	0.18
Female (%)	330 (60.89)	73 (81.11)	<0.001
Median years of education (IQR)	17.00 (4.00)	16.00 (6.00)	0.43
APOEε4+ (%)	192 (35.42)	43 (47.78)	0.03
Parental dementia history (%)	290 (53.51)	37 (41.11)	0.03
Ethnicity: Caucasian (%)	524 (98.50)	81 (90.00)	0.004
Median alcohol units per week (IQR)	9.00 (12.00)	8.00 (8.00)	0.19
Mean age onset IPV-PA, years (SD)	–	20.70 (11.80)	–
Mean duration of IPV-PA, years (SD)	–	6.57 (7.83)	–
Median time since IPV-PA ended, years (IQR)	–	27.00 (14.00)	–

APOE, apolipoprotein E; IPV-PA, intimate partner violence-related physical abuse.;

### Intimate partner violence-related physical abuse and adverse mental health in midlife

Risk of both lifetime and ongoing mental health disorders were higher among participants reporting IPV-PA than among those reporting no exposure (table 2). Thus, in fully adjusted models, compared with participants with no exposure, history of IPV-PA was associated with higher risk of ongoing depression (adjusted OR (aOR) 2.92; 95% CI 1.54 to 5.35; p<0.001), anxiety (aOR 3.74; 95% CI 1.97 to 6.89; p<0.001), sleep disorder (aOR 2.44; 95% CI 1.38 to 4.22; p=0.002) and PTSD symptomology (aOR 4.34; 95% CI 2.73 to 6.92; p<0.001), with risk of concurrent mental health disorders also higher among those with IPV-PA (incidence rate ratio (IRR) 2.31; 95% CI 1.82 to 2.91; p<0.001) (online supplemental figure 1). However, in a sensitivity analysis excluding those who reported a history of PA before the age of 16 years, the observed increased lifetime risk of depression, as well as ongoing and lifetime sleep disorder, was attenuated, while all other relationships remained (online supplemental table 1).

**Table 2:** Associations between IPV-PA and lifetime and ongoing mental health outcomes

	No exposure (n=542)	IPV-PA(n=90)	aOR* (95% CI)	P value
Depression				
Lifetime (%)	129 (23.80)	34 (37.78)	1.93 (1.20 to 3.08)	0.006
Ongoing (%)	40 (7.38)	17 (18.89)	2.92 (1.54 to 5.35)	<0.001
Anxiety				
Lifetime (%)	70 (12.92)	31 (34.44)	3.54 (2.13 to 5.82)	<0.001
Ongoing (%)	34 (6.27)	18 (20.00)	3.74 (1.97 to 6.89)	<0.001
Sleep disorder				
Lifetime (%)	66 (12.18)	23 (25.56)	2.50 (1.43 to 4.24)	<0.001
Ongoing (%)	60 (11.07)	21 (23.33)	2.44 (1.38 to 4.22)	0.002
PTSD symptomology				
Lifetime (%)	239 (44.10)	78 (86.67)	8.24 (4.55 to 16.25)	<0.001
Ongoing (%)	113 (20.85)	48 (53.33)	4.34 (2.73 to 6.92)	<0.001

* All models adjusted for age, gender, education and TBI with LOC. Models for depression, anxiety and sleep disorders also included adjustment for PTSD symptomology.

aOR, adjusted OR; IPV-PA, intimate partner violence-related physical abuse; LOC, loss of consciousness; PTSD, post-traumatic stress disorder; TBI, traumatic brain injury.

### Traumatic brain injury is common among individuals reporting IPV-PA and associated with specific mental health outcomes

Among participants with history of IPV-PA, 79 out of 90 (87.78%) reported a history of blows to the head, of which 64 (71.11%) reported multiple blows, with TBI exposures higher among participants with IPV-PA than among participants with no exposure. Specifically, compared with participants with no exposure, those with a history of IPV-PA reported a higher number of head injuries with LOC (p=0.003), and head injuries with DAC (p=0.001). Among participants reporting head injuries with LOC, the overwhelming majority reported <30 min LOC, consistent with mild TBI^20^ (95.65% among those with a history of IPV-PA and 95.93% among those without IPV-PA exposure) (table 3).

**Table 3:** Lifetime TBI characteristics

	No exposure(n=542)	IPV-PA(n=90)	P value
Blows to head, n (%)	393 (72.51)	79 (87.78)	0.002
Multiple blows to head, n (%)	287 (52.95)	64 (71.11)	0.001
Total number blows to head, n (IQR)	2.00 (4.00)	3.00 (8.00)	<0.001
LOC, n (%)	172 (31.73)	46 (51.11)	<0.001
Episodes LOC, n (IQR)	0.00 (1.00)	1.00 (2.00)	0.003
Maximum LOC <30 min (mTBI), n (%)	165 (95.93)	44 (95.65)	–
DAC, n (%)	250 (46.13)	61 (67.78)	<0.001
Episodes DAC, n (IQR)	1.00 (2.00)	3.47 (2.00)	0.001
Maximum DAC <30 min, n (%)	33 (13.20)	11 (18.03)	0.04

DAC, dazed and confused following blow to head; IPV-PA, intimate partner violence-related physical abuse; LOC, loss of consciousness following blow to head; mTBI, mild traumatic brain injury.

In individuals with a history of IPV-PA, TBI with LOC was associated with a considerable increased risk of PTSD and of complex, mixed mental health outcomes. Thus, TBI with LOC was associated with increased risk of both lifetime (OR 6.49, 95% CI 1.51 to 45.58; p=0.02) and ongoing (OR 5.07, 95% CI 2.00 to 13.82; p<0.001) PTSD symptomology and a higher number of concurrent, ongoing mental health conditions (IRR 1.81, 95% CI 1.21 to 2.76; p=0.005). There were, however, no associations between duration of IPV-PA or time since abuse ended and the remaining outcome measures (table 4). In contrast, in the absence of IPV-PA, history of TBI was only associated with higher lifetime risk of PTSD (aOR 1.74, 95% CI 1.19 to 2.54; p*=*0.04), although at a lower level than in those reporting TBI with IPV-PA (online supplemental table 2).

**Table 4:** Associations between IPV-PA variables and lifetime and ongoing mental health outcomes

	Lifetime		Ongoing	
	aOR* (95% CI)	**P value**	aOR* (95% CI)	**P value**
Depression				
TBI with LOC	2.34 (0.94 to 6.05)	0.07	2.12 (0.67 to 7.48)	0.21
Age	0.98 (0.90 to 1.07)	0.67	1.00 (0.90 to 1.12)	0.94
Years since IPV-PA	0.98 (0.93 to 1.03)	0.35	0.97 (0.92 to 1.03)	0.39
Duration of IPV-PA	1.03 (0.97 to 1.09)	0.37	1.01 (0.95 to 1.08)	0.67
Anxiety				
TBI with LOC	1.20 (0.48 to 3.08)	0.70	1.79 (0.58 to 5.88)	0.32
Age	1.04 (0.95 to 1.15)	0.36	1.04 (0.94 to 1.16)	0.46
Years since IPV-PA	0.99 (0.94 to 1.04)	0.77	0.97 (0.91 to 1.03)	0.26
Duration of IPV-PA	1.03 (0.97 to 0.09)	0.29	1.00 (0.93 to 1.07)	0.96
Sleep disorder				
TBI with LOC	1.56 (0.54 to 4.69)	0.42	1.71 (0.58 to 5.29)	0.34
Age	1.14 (1.01 to 1.30)	0.04	1.11 (0.99 to 1.26)	0.10
Years since IPV-PA	1.02 (0.96 to 1.08)	0.59	1.02 (0.96 to 1.08)	0.55
Duration of IPV-PA	1.03 (0.96 to 1.10)	0.38	1.01 (0.93 to 1.08)	0.81
PTSD				
TBI with LOC	6.49 (1.51 to 45.58)	0.02	5.07 (2.00 to 13.82)	<0.001
Age	0.98 (0.84 to 1.12)	0.73	0.96 (0.87 to 1.05)	0.34
Years since IPV-PA	1.01 (0.92 to 1.09)	0.83	1.04 (0.99 to 1.10)	0.11
Duration of IPV-PA	1.03 (0.94 to 1.15)	0.56	1.01 (0.96 to 1.08)	0.64
	**LifetimeIRR (95% CI**)	**P value**	**OngoingIRR (95% CI**)	**P value**
Total conditions				
TBI with LOC	1.36 (1.00 to 1.88)	0.53	1.81 (1.21 to 2.76)	0.005
Age	1.02 (0.99 to 1.05)	0.26	1.02 (0.98 to 1.06)	0.28
Years since IPV-PA	1.00 (0.98 to 1.01)	0.75	1.00 (0.98 to 1.02)	0.90
Duration of IPV-PA	1.01 (0.99 to 1.03)	0.26	1.01 (0.98 to 1.03)	0.56

* All models adjusted for age, gender and education. Models for depression, anxiety and sleep disorders also included adjustment for PTSD symptomology.

aOR, adjusted OR; IPV-PA, intimate partner violence-related physical abuse; IRR, incidence rate ratio; LOC, loss of consciousness; PTSD, post-traumatic stress disorder; TBI, traumatic brain injury.

## Discussion

Data from this ongoing, prospective, longitudinal study of midlife indices of brain health demonstrate that around 14% of participants reported a history of IPV-PA at baseline assessment, a majority of these were female. As anticipated, histories of repetitive blows to the head and TBI were frequent among those reporting IPV-PA. Notably, among participants reporting IPV-PA, the risk of lifetime and ongoing adverse mental health disorders remained high, despite the abuse typically having ceased many years prior to the study. Specifically, compared with participants reporting no IPV-PA, those with a history of IPV-PA had a higher risk of ongoing depression, anxiety, sleep disorder, PTSD symptomatology and concurrent mental health disorders. Intriguingly, a history of TBI with LOC was associated with both lifetime and ongoing symptoms of PTSD, as well as concurrent, ongoing mental health conditions in midlife.

Existing literature from WHO suggests that, globally, up to 30% of women will experience physical or sexual IPV in their lifetime,^5^ with UK-specific data reporting a prevalence of 24%.^21^ Our finding that 14.2% of participants in our cohort reported IPV-PA may appear inconsistent with these international and nationally relevant figures. However, our data do not include participants whose exposure may have been limited to sexual IPV, as this is not currently captured in the PREVENT datasets. Moving forward, IPV data collection in this cohort—and in broader longitudinal research on brain health—could be strengthened by the introduction of a suitable, standardised tool that captures comprehensive, lifetime IPV exposures. Nevertheless, consistent with previous reporting,^10^ we observed that the overwhelming majority of those exposed to IPV-PA in our cohort were women. These findings underscore the significant public health impact of IPV, which carries an estimated cost to society exceeding £78 billion annually in England and Wales alone.^22^

There have been several reports documenting adverse mental health outcomes in association with IPV, including higher rates of depression, anxiety and PTSD.^13 23 24^ Typically, however, these studies report mental health outcomes in participants either actively exposed to abuse or in the years immediately after^25 26^ and, most often, in younger women before midlife.^27 28^ They are further limited in terms of generalisability due to typically small sample sizes and restricted populations (eg, military personnel and women’s shelters).^23 24 29^ The PREVENT Dementia cohort comprises research volunteers aged 40–59 years at recruitment and was established to profile midlife risk factors for later-life neurodegeneration to identify earliest indicators of NDD.^16^ It was not designed, therefore, as a specific study recruiting for history of IPV, but as a cross-section of the population in the UK and Ireland. Nevertheless, our data demonstrate a higher risk of lifetime and ongoing diagnoses of various mental health disorders among individuals with IPV-PA exposure compared with those without such exposure. Importantly, among these participants, the risk of lifetime and ongoing mental health disorders was independent of the time since abuse ended, indicating that the increased risk observed was not driven by those with more recent exposures. However, in a sensitivity analysis excluding those reporting onset of abuse prior to age 16 years, the relationship between IPV-PA and risk of lifetime, but not ongoing, depression, and risk of both lifetime and ongoing sleep disorder was attenuated. Further studies exploring this intriguing age-dependent relationship would be informative.

As anticipated, the prevalence of repetitive blows to the head and mild TBI among our participants reporting a history of IPV was high.^20^ These findings are consistent with existing research in this area reporting high prevalence of head and neck injury in IPV,^7 8 10^ often with associated repeated TBI exposure.^8 30^ There is considerable evidence linking a history of TBI to adverse brain health outcomes.^1^,^4^ With regard to mental health disorders, a history of TBI—regardless of severity—is associated with an increased risk of anxiety and depression, typically in the years immediately following injury.^3 4^ Intriguingly, female sex is often documented among risk factors for the development of mental health disorders following TBI.^3^ However, many of these studies do not report the mechanism of injury, particularly whether it was attributable to IPV-PA. In contrast, our data show that among individuals with histories of IPV-PA, only PTSD symptomology and concurrent mental health disorders in midlife were associated with TBI exposure. In part, this might reflect our cohort having been exposed to IPV-PA many years prior to assessment; studies typically report higher risk of wider mental health outcomes in the years immediately following TBI. Notably, although the difference did not reach statistical significance, lifetime risk of depression was higher among participants with IPV-PA. Therefore, despite our relatively large study population and comparisons with non-exposed individuals, further research involving larger cohorts is needed to explore these specific relationships more thoroughly.

Nevertheless, as noted, we observed that TBI among individuals exposed to IPV-PA was associated with an increased risk of both ongoing and lifetime symptoms of PTSD. This finding is consistent with previous reports of PTSD symptoms associated with recent (within 1 year) IPV with TBI exposure in a small cohort recruited from women’s shelters,^30^ as well as in a cohort of female military veterans with lifetime histories of IPV and TBI.^23^ Our finding of persisting PTSD symptoms in a midlife community cohort, many years after experiencing IPV-PA and TBI, highlights the potential lifelong consequences of such exposure. Given the prevalence of IPV in society—often accompanied by TBI, as observed in this study—there are clearly considerable implications for adult mental health services.

While providing important insights into the potential later-life mental health implications of IPV-PA and TBI, our current study design does have limitations that should be addressed in future research. Accessing data from an established, ongoing, longitudinal research programme exploring midlife brain health offers key strengths, particularly the richness of available data within a dedicated research volunteer population. However, as the PREVENT study was not originally designed with IPV in mind, there are limitations in the data collected. Specifically, although partner violence is the predominant form of interpersonal violence experienced by women, we cannot exclude the possibility that instances of violence from non-partners may have been included in response to the questions posed in the Life Stressor Checklist. Furthermore, instances of PA in the context of sexual violence—such as choking—may be under-reported using our approach. While the Life Stressor Checklist-Revised includes a screening question that can be interpreted as an indicator of IPV-PA, it is less robust than dedicated tools specifically designed for identifying IPV. Additionally, by capturing the duration of exposure as the time from first to last incident, rather than duration of each individual episode, the questionnaire may potentially overestimate the total lifetime duration of IPV-PA exposure in cases involving multiple, intermittent instances. As a result, the influence of IPV-PA duration on outcomes may be underestimated. Further data collection in this cohort—and in broader IPV research—would benefit from the application of more robust IPV and IPV-related TBI screening tools, such as those recommended by the ENIGMA IPV working group.^6^ Finally, reflecting the demographic profile of volunteers participating in research at PREVENT centres, our study population is overwhelmingly Caucasian. As such, the generalisability of findings to more diverse populations—where the profiles of IPV and mental health disorders may differ—remains unknown.

In summary, our data demonstrate that in this general population cohort recruited at midlife for longitudinal studies of brain health, history of IPV-PA was more common among female participants and was associated with evidence of persistent and later-onset adverse mental health outcomes. Furthermore, among those reporting exposure to IPV-PA, the majority reported a history of repetitive blows to the head and mild TBI. A history of TBI, in turn, was associated with evidence of adverse mental health outcomes. These observations offer initial insights into the potential lifelong brain health consequences of IPV. Given the global prevalence of IPV, particularly among women, these findings highlight a pressing need for further research in this field, as well as for targeted interventions to raise awareness among policymakers and medical professionals about the potential impact of IPV on health outcomes across the lifespan.

## Supplementary material

10.1136/bmjment-2024-301439 online supplemental file 1

10.1136/bmjment-2024-301439 online supplemental file 2

## Data Availability

Data are available on reasonable request.
