# Supplementary material for: Intimate partner violence, traumatic brain injury and long-term mental health outcomes in midlife: the Drake IPV study
Source: BMJ Ment Health. 2025 Jun 9;28(1):e301439. doi: 10.1136/bmjment-2024-301439 (PMC12161325; doi:10.1136/bmjment-2024-301439)
Supplement: online supplemental file 1 [file bmjment-28-1-s001.docx]

**Intimate partner violence, traumatic brain injury and long-term mental health outcomes in mid-life: The Drake IPV study.**

Natalie D Jenkins, Craig W Ritchie, Karen Ritchie, Graciela Muniz-Terrera, William Stewart and the PREVENT Dementia Investigators

**Contents:**

**Page 2 Supplementary Figure 1:** Percentage of participants with number of ongoing mental health conditions.

**Page 3 Supplementary Table 1:** Sensitivity analysis excluding participants reporting abuse aged under 16 years from analyses.

**Page 4 Supplementary Table 2:** Associations between history of TBI with loss of consciousness in individuals with no IPV-PA exposure and lifetime and ongoing mental health outcomes

**
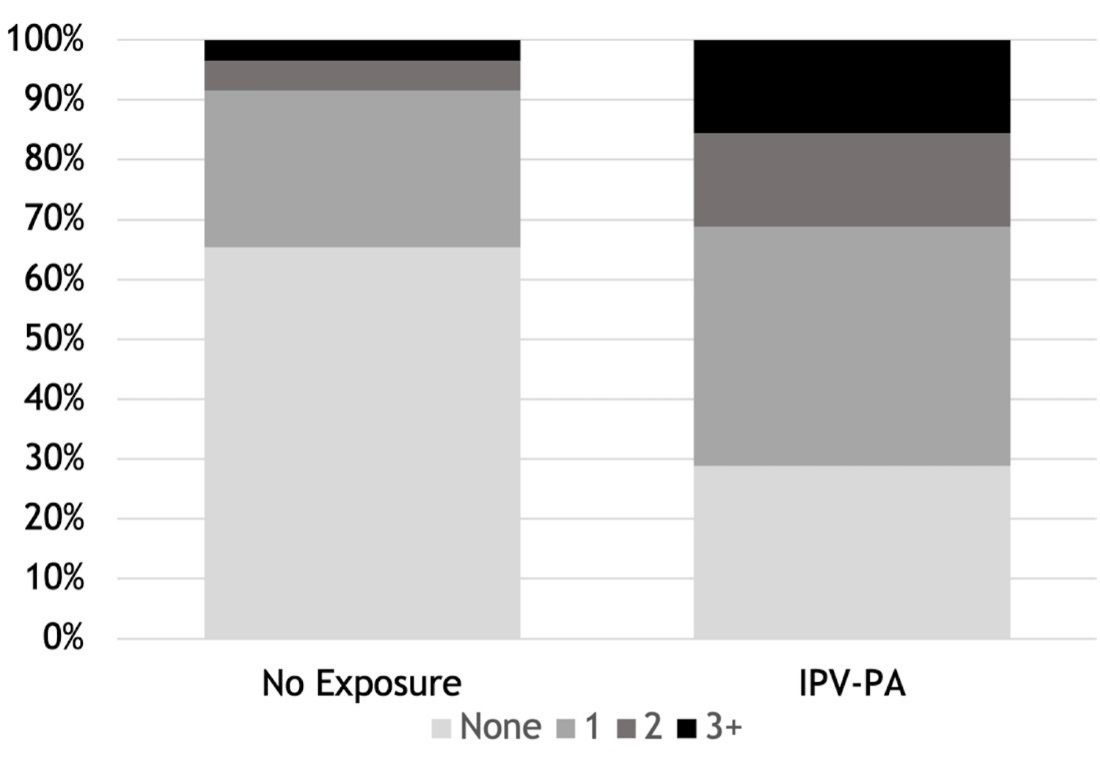
**

**Figure 1:** Percentage of participants with number of ongoing mental health conditions. IPV-PA was asociated with a higher risk of concurrent conditions (two or more ongoing conditions) in mid-life compared to participants with no exposure (RR 2.31; 95%CI 1.82-2.91; p<0.001).

**Supplementary Table 1:** Sensitivity analysis excluding participants reporting abuse aged under 16 years from analyses.

|  | **Lifetime** | | **Active/Ongoing** | |
| --- | --- | --- | --- | --- |
|  | **aOR* (95% CI)** | **p-value** | **aOR* (95% CI)** | **p-value** |
| **Depression** | 1.39 (0.74-2.57) | 0.30 | 2.44 (1.07 – 5.29) | 0.03 |
| **Anxiety** | 2.50 (1.28 – 4.77) | 0.006 | 3.41 (1.54 – 7.30) | 0.001 |
| **Sleep disorder** | 1.51 (0.68 – 3.12) | 0.28 | 1.43 (0.63 – 3.03) | 0.37 |
| **PTSD Symptomology** | 7.36 (3.54 – 17.30) | <0.001 | 2.99 (1.66 – 5.34) | <0.001 |

aOR, Adjusted odds ratio; CI, confidence interval; IRR, incidence rate ratio; IPV-PA, Intimate partner violence related physical abuse; PTSD, post-traumatic stress disorder. *all models adjusted for age, gender, education, and TBI with LOC. Models for depression, anxiety, and sleep disorders also included adjustment for PTSD symptomology.

**Supplementary Table 2:** Associations between history of TBI with loss of consciousness in individuals with no IPV-PA exposure and lifetime and ongoing mental health outcomes.

|  | **Lifetime**  **aOR* (CI)** | ***p*-value** | **Ongoing**  **aOR* (CI)** | ***p*-value** |
| --- | --- | --- | --- | --- |
| **Depression** | 1.01 (0.64 – 1.58) | 0.96 | 1.06 (0.50 – 2.15) | 0.87 |
| **Anxiety** | 0.89 (0.49 – 1.56) | 0.69 | 0.77 (0.32 – 1.70) | 0.53 |
| **Sleep disorder** | 0,99 (0.55 – 1.74) | 0.97 | 0.98 (0.53 – 1.76) | 0.94 |
| **PTSD** | 1.74 (1.19 – 2.54) | 0.004 | 1.35 (0.85 – 2.12) | 0.19 |
|  | **Lifetime**  **IRR (95%CI)** | ***p*-value** | **Ongoing**  **IRR (95%CI)** | ***p*-value** |
| **Total conditions** | 1.16 (0.96 – 1.40) | 0.13 | 1.15 (0.88 – 1.50) | 0.30 |

aOR, Adjusted odds ratio; CI, confidence interval; IRR, incidence rate ratio; PTSD, post-traumatic stress disorder. *all models adjusted for age, gender, and education. Models for depression, anxiety, and sleep disorders also included adjustment for PTSD symptomology.
