# Supplementary material for: Intimate partner violence, traumatic brain injury and long-term mental health outcomes in midlife: the Drake IPV study
Source: BMJ Ment Health. 2025 Jun 9;28(1):e301439. doi: 10.1136/bmjment-2024-301439 (PMC12161325; doi:10.1136/bmjment-2024-301439)
Supplement: online supplemental file 2 [file bmjment-28-1-s002.docx]

| Name | Affiliation |
| --- | --- |
| Katie Bridgeman | Edinburgh Dementia Prevention, University of Edinburgh, Edinburgh, UK |
| Dr Leonidas Chouliaras | Department of Psychiatry, University of Cambridge School of Clinical Medicine, Cambridge, UK |
| Siobhan Coleman | Windsor Research Unit, Cambridgeshire and Peterborough NHS Foundation Trust, Cambridge, UK |
| Hannah Darwin | Edinburgh Dementia Prevention, University of Edinburgh, Edinburgh, UK |
| Dr Maria-Eleni Dounavi | Department of Psychiatry, University of Cambridge School of Clinical Medicine, Cambridge, UK |
| Feng Deng | Global Brain Health Institute, Trinity College Dublin, Dublin, Ireland |
| Dr Robert Dudas | Cambridgeshire and Peterborough NHS Foundation Trust, Cambridge, UK |
| Dr Sarah Gregory | Scottish Brain Sciences, Edinburgh, UK  Edinburgh Dementia Prevention, University of Edinburgh, Edinburgh, UK |
| Dr Ivan Koychev | Department of Psychiatry, University of Oxford, Oxford, UK |
| Prof Brian Lawlor | Global Brain Health Institute, Trinity College Dublin, Dublin, Ireland |
| Dr Audrey Low | Department of Psychiatry, University of Cambridge School of Clinical Medicine, Cambridge, UK |
| Prof Clare Mackay | Department of Psychiatry, University of Oxford, Oxford, UK |
| Dr Elijah Mak | Department of Psychiatry, University of Cambridge School of Clinical Medicine, Cambridge, UK |
| Prof Paresh Malhotra | Department of Brain Sciences, Imperial College London, London, UK |
| Prof Graciela Muniz-Terrera | Edinburgh Dementia Prevention, University of Edinburgh, Edinburgh, UK  Ohio University Heritage College of Osteopathic Medicine, Ohio University, Ohio, |
| Prof Lorina Naci | School of Psychology and Global Brain Health Institute, Trinity College Dublin, Dublin, Ireland |
| Prof John O’Brien | Department of Psychiatry, University of Cambridge School of Clinical Medicine and Cambridgeshire and Peterborough NHS Foundation Trust, Cambridge, UK |
| Dr Vanessa Raymont | Department of Psychiatry, University of Oxford, Oxford, UK |
| Prof Craig Ritchie | Scottish Brain Sciences, Edinburgh, UK  Edinburgh Dementia Prevention, University of Edinburgh, Edinburgh, UK  Mackenzie Institute, St Andrews University, St Andrews, UK |
| Professor Li Su | Department of Psychiatry, University of Cambridge School of Clinical Medicine, Cambridge, UK |
| Dr Peter Swann | Department of Psychiatry, University of Cambridge School of Clinical Medicine, Cambridge, UK and Cambridgeshire and Peterborough NHS Foundation Trust, Cambridge, UK |
| Tony Thayanandan | Department of Psychiatry, University of Oxford, Oxford, UK |
| Prof Guy Williams | Wolfson Brain Imaging Centre and Department of Clinical Neurosciences, University of Cambridge School of Clinical Medicine, Cambridge, UK |
